# Material-driven fibronectin and vitronectin assembly enhances BMP-2 presentation and osteogenesis

**DOI:** 10.1016/j.mtbio.2022.100367

**Published:** 2022-07-19

**Authors:** Yinbo Xiao, Hannah Donnelly, Mark Sprott, Jiajun Luo, Vineetha Jayawarna, Leandro Lemgruber, P. Monica Tsimbouri, R.M. Dominic Meek, Manuel Salmeron-Sanchez, Matthew J. Dalby

**Affiliations:** a Centre for the Cellular Microenvironment, Institute of Molecular, Cell & Systems Biology, College of Medical, Veterinary and Life Sciences, Joseph Black Building, University of Glasgow, Glasgow, G12 8QQ, United Kingdom; b Centre for the Cellular Microenvironment, Division of Biomedical Engineering, School of Engineering, University of Glasgow, Glasgow, G12 8QQ, United Kingdom; c Glasgow Imaging Facility, Institute of Infection, Immunity and Inflammation, College of Medical, Veterinary and Life Sciences, University of Glasgow, Glasgow, G12 8TA, United Kingdom; d Department of Trauma and Orthopaedics, Queen Elizabeth University Hospital, Glasgow, G51 4TF, UK

**Keywords:** Mesenchymal stem cells, Polymer organized extracellular matrix, Vitronectin, Fibronectin, Bone morphogenetic protein-2, Osteogenesis

## Abstract

Mesenchymal stem cell (MSC)-based tissue engineering strategies are of interest in the field of bone tissue regenerative medicine. MSCs are commonly investigated in combination with growth factors (GFs) and biomaterials to provide a regenerative environment for the cells. However, optimizing how biomaterials interact with MSCs and efficiently deliver GFs, remains a challenge. Here, via plasma polymerization, tissue culture plates are coated with a layer of poly (ethyl acrylate) (PEA), which is able to spontaneously permit fibronectin (FN) to form fibrillar nanonetworks. However, vitronectin (VN), another important extracellular matrix (ECM) protein forms multimeric globules on the polymer, thus not displaying functional groups to cells. Interestingly, when FN and VN are co-absorbed onto PEA surfaces, VN can be entrapped within the FN fibrillar nanonetwork in the monomeric form providing a heterogeneous, open ECM network. The combination of FN and VN promote MSC adhesion and leads to enhanced GF binding; here we demonstrate this with bone morphogenetic protein-2 (BMP2). Moreover, MSC differentiation into osteoblasts is enhanced, with elevated expression of osteopontin (OPN) and osteocalcin (OCN) quantified by immunostaining, and increased mineralization observed by von Kossa staining. Osteogenic intracellular signalling is also induced, with increased activity in the SMAD pathway. The study emphasizes the need of recapitulating the complexity of native ECM to achieve optimal cell-material interactions.

## Highlights

• Vitronectin can be incorporated within fibronectin fibril networks upon co-coating onto poly (ethyl acrylate) modified surfaces.
• Fibronectin and vitronectin networks promote mesenchymal stem cell adhesion and induce α5 integrin clustering.
• Fibronectin and vitronectin nanonetworks improve bone morphogenetic protein-2 presentation to mesenchymal stem cells and thus facilitates osteogenesis.

## Graphical abstract

**Figure:**
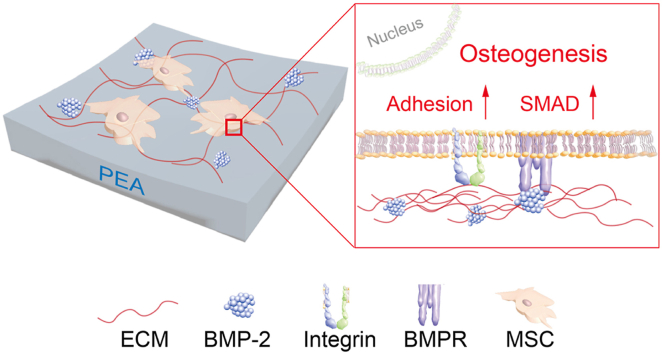

## CRediT author statement

**Yinbo Xiao**: Methodology, Formal analysis, Investigation, Writing – Original Draft; **Hannah Donnelly**: Data Curation, Writing – Review & Editing, Supervision; **Mark Sprott**: Visualization, Investigation; **Jiajun Luo**: Visualization; **Vineetha Jayawarna**: Data Curation, Visualization; **Leandro Lemgruber**: Visualization; **P. Monica Tsimbouri**: Methodology; **Robert M.D. Meek**: Resources; **Manuel Salmeron-Sanchez**: Resources, Supervision; **Matthew J. Dalby**: Conceptualization, Writing – Review & Editing, Supervision, Funding acquisition.

## 1 Introduction

Biomaterials in bone tissue engineering have gained popularity as a way to aid bone regeneration in response to disease, trauma, or injury. Growing biomaterials are developed to enhance initial stem cells’ adhesion, and subsequently, drive their differentiation into osteoblasts, thus promoting bone regeneration [1]. However, often for cells to adhere effectively to these materials they must first organise extracellular matrix (ECM) proteins absorbed onto the material surface. The presentation of ECM ligands ultimately affects how cells sense such microenvironmental stimuli through revealing cryptic sites that cells can adhere to or that growth factors can absorb onto [2]. Further to this, these material-based approaches do not typically control growth factor (GF) presentation or GF-ECM binding [3]. This commonly means that where GFs are required, such as in bone morphogenetic protein 2 (BMP-2) in bone regeneration, uncontrolled, soluble delivery of GFs at supraphysiological levels is employed [4]. Therefore, the development of materials that exploit ECM proteins to reveal cell adhesion and GF binding sites, that can be decorated with low, but effective loads of GFs may provide a better approach for controlling stem cell differentiation and improving the translation of these systems to the clinic.

ECM proteins play a pivotal role in modulating many cellular processes, such as adhesion, migration and differentiation [2]. Once attached, cell membrane receptors such as integrins, can recognise and couple to the cell-binding domains of ECM proteins [5]. Such integrin-ECM couplings can transduce extracellular ECM signals via the actin cytoskeleton, regulating related gene transcriptional activities and thus directing cell behaviours [5]. ECM proteins containing heparin-II binding sites have been shown to bind several families of GFs with high affinity [6]. As such, the complex ECM network serves as a GF reservoir, releasing GFs in a sustained manner to the local microenvironment and thus orchestrating cell biological processes [6,7]. It should be noted that the availability of these critical functional domains depends on the ECM protein conformation. Folded or globular protein conformations can hide these functional domains rendering them unavailable for interaction, limiting their biological actions and utilization for tissue bioengineering [8,9].

Polymers are currently being investigated as a versatile and potent tool in the field of tissue engineering. Certain polymers allow precise control of the physical and chemical properties of the materials, such as mechanical properties, topographical features and chemical composition [9]. By optimizing surface chemical modification, our previous work has shown that poly (ethyl acrylate) (PEA) could spontaneously drive fibronectin (FN) into fibrillar-like networks, which physiologically mimics their *in vivo* conformation [10,11]. This fibrillar conformation can expose the key functional domains of FN, e.g. heparin-II GF binding domain and the RGD (Arginine-Glycine-Aspartate) integrin-binding domain, subsequently allowing control of GF delivery and cellular adhesion, and ultimately cellular differentiation [11]. Importantly, ultra-low doses of GFs such as bone morphogenetic protein-2 (BMP2), and vascular endothelial growth factor (VEGF), have previously been shown to successfully bind to the FN fibrillar network and elicit cell-type-specific responses [[12], [13], [14]]. As such, PEA has been successfully translated to *in vivo* studies and veterinary trials on large bone defects with promise for both osteogenic and angiogenic outcomes [[12], [13], [14]].

Besides FN, vitronectin (VN) is another critical component within the ECM network, serving the function of being a biological ECM glue [15]. In contrast to the structural functions of other ECM proteins, VN serves as a matricellular protein, whose functions are achieved by binding to other matrix proteins and thus form an interconnected ECM network [16]. Its biological functions are dependent on the conformation of its secondary structure. Upon undergoing a conformational change from monomeric form to multimeric form, VN becomes more biologically active by exposing its heparin-binding and cell-binding sites [[17], [18], [19]]. In particular, the cellular αV integrins are shown to couple with cell-binding sites of VN [20]. Interestingly, unlike FN-binding α5 integrins, αV integrins are mostly shown to confine to the cell edge and exhibit a weaker bond force to ECM [21,22]. It is of note that cells are not able to drive FN fibrillogenesis in the absence of VN-αV integrin coupling [22]. These findings suggest that the synergistic control among integrin-ECM coupling affects cell behaviours and ECM remodelling. Our previous work has already shown that VN activity is dependent on the surface chemical composition [23,24]. By changing the amount of hydroxyl groups to increase surface hydrophobicity, the bioactivity of VN was increased, leading to increased cell spreading and focal adhesion development in cells [25]. Moreover, coinciding with the previous study, the presence of VN can increase FN assembly and enhance cell-mediated FN reorganization and secretion [23,24].

Previous studies have demonstrated that PEA can spontaneously drive absorbed FN into biological nanonetworks [13,14]. Further, when VN is mixed with FN, nanonetworks are also seen and enhance cell adhesion [[22], [23], [24]]. However, these studies don't clarify the ability of these blended FN/VN networks to interact with GFs, such as BMP-2, and to direct MSC fates. In this new study, by adding BMP2 into such a blended ECM network, we demonstrate that the FN ​+ ​VN ECM network can bind increased levels of BMP-2 and effectively present it to MSCs, thus directing osteogenesis. Based on the plasma polymerization of PEA [26], we explore the effects of FN and VN on α5/αV integrin ligation. As has been shown before [23], VN was observed to be within PEA-driven FN fibril networks and the presence of VN promoted cell spreading and adhesion. Then the GF BMP2, widely used in bone tissue engineering [27,28], was incorporated into these ECM networks and the osteogenesis of MSCs was evaluated. We found this complex ECM composite could bind increased levels of BMP2 and effectively present it to MSCs, thus directing robust osteogenesis. These findings shed light on the important role of ECM complexity *in vitro*.

## 2 Materials and methods

### 2.1 Plasma polymerization

**Fig. 1:**
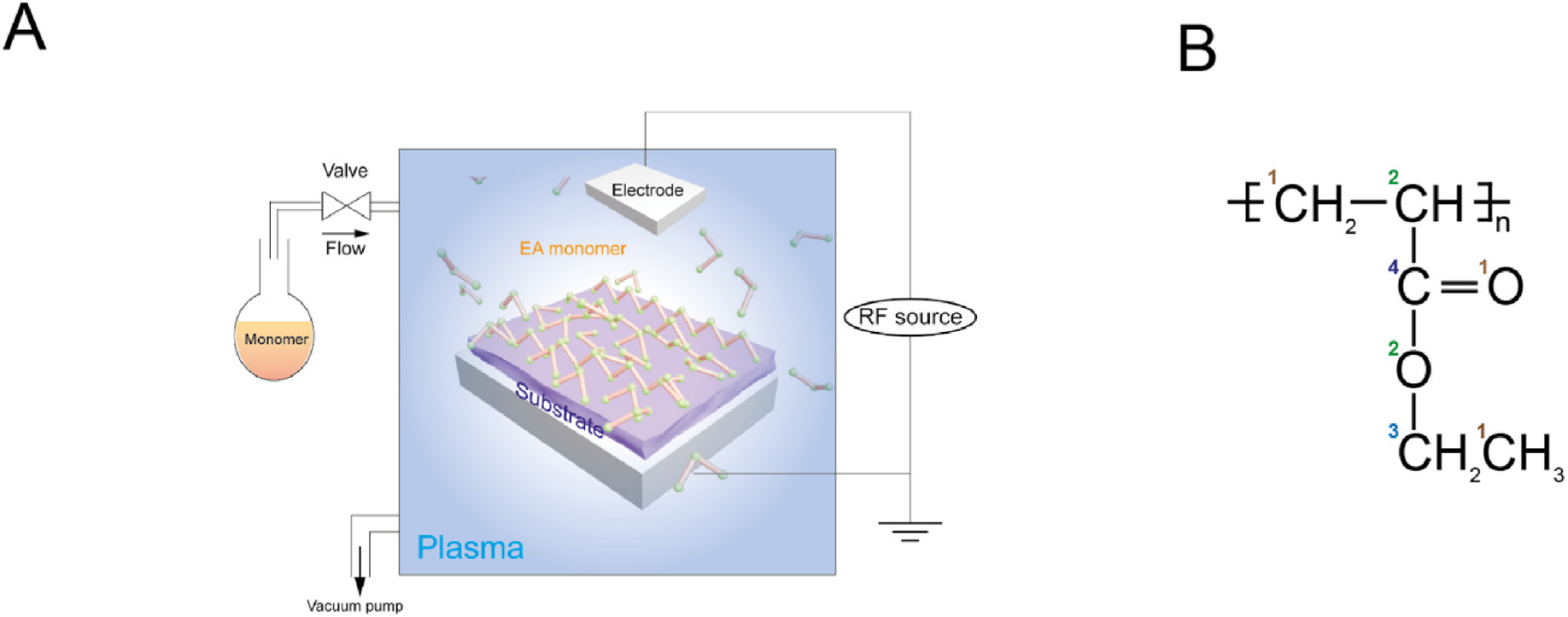
Plasma equipment setup and chemical structure of PEA. (A), Schematic representation of a customized plasma polymerization chamber. (B), Chemical structure of PEA. The numbers in different colours correspond to the different chemical bonds. (For interpretation of the references to colour in this figure legend, the reader is referred to the Web version of this article.)

Plasma equipment was set up according to our previous work (Fig. 1A) [13,26]. A custom-built plasma reactor was used to polymerise ethyl acrylate (EA) via plasma polymerization. The EA plasma was generated by two capacitively coupled copper band electrodes which connected to a radio frequency power supply. Details of other design and operation considerations to facilitate the polymerization of EA can be found in our previous study [13,26]. The chemical structure of PEA was shown in Fig. 1B. Briefly, samples (Glass coverslips, Nunc™ Thermanox™ coverslips or Commercial Corning® 48-well tissue culture plates (TCP)) were placed in the plasma chamber vertically to the plasma flow. Then, samples were exposed to air plasma for 5 ​min at 50 ​W of radio frequency (RF) incident power to ensure the removal of any residual organic matter. As for the PEA plasma, the RF power applied to the plasma chamber was set as 50 ​W, and the plasma treatment time was set as 15 ​min. Before the ECM coating for cell culture, the samples were sterilized under ultraviolet (UV) light for 30 ​min.

### 2.2 Water contact angle (WCA) measurement

Water contact angle (WCA) measurements were taken on PEA-coated glass coverslips pre and post-UV treatment. Static contact angles (SCAs) were measured by dropping a 3 ​μL drop of deionised water onto the surfaces using a Theta optical tensiometer (Biolin Scientific, Stockholm, Sweden) (n ​= ​12).

### 2.3 X-ray photoelectron spectra (XPS) analysis

After plasma polymerization, samples were sent out to the National EPSRC Users’ Service (NEXUS) (found at: http://www.ncl.ac.uk/nexus/) for X-ray photoelectron spectra (XPS) analysis. A K-alpha XPS system (ThermoScientific) equipped with a monochromatic Al K-alpha source was used to analyse each sample three times at a maximum beam size of 400 ​μm ​× ​800 ​μm. Parameters were set up as follows: X-ray energy: 1486.68 ​eV; voltage: 12 ​kV; current: 3 ​mA; power: 36 ​W. Spectra analysis and curve fitting were performed using CasaXPS software.

### 2.4 ECM and BMP2 adsorption

For ECM protein adsorption, phosphate-buffered saline (PBS) solution containing human FN (20 ​μg ​ml^−1^) (Sigma-Aldrich, #F2006), human VN (20 ​μg ​ml^−1^) (Sigma-Aldrich, #5051), or the blend of FN (10 ​μg ​ml^−1^) and VN (10 ​μg ​ml^−1^) were coated onto PEA modified TCPs for 1 ​h at room temperature, termed as FN, VN and FN ​+ ​VN respectively. Following PBS washes, samples were ready for cell culture. For GF adsorption, BMP2 (Sigma-Aldrich, #H4791) was diluted with PBS (final concentration 50 ​ng ​ml^−1^) and incubated onto ECM-coated TCPs for 2 ​h at room temperature, followed by washing with PBS. TCPs without plasma PEA treatment were used as the control substrate.

### 2.5 Atomic Force Microscopy (AFM)

ECM protein conformation images were obtained using Atomic Force Microscopy (AFM). Before ECM protein absorption, PEA-treated glass coverslips were imaged by AFM as the control. After ECM protein absorption, substrates were washed with PBS, and then finally with deionised water. The samples were then dried with nitrogen gas and allowed for further air-drying before AFM imaging. The JPK Nanowizard 4 was used for imaging and the JPK Data Processing software version 5 was used for image analysis. A pyramidal silicon tip was utilised for alternating contact mode imaging, with a resonance frequency of 70 ​kHz and stiffness of 3 ​N ​m^−1^.

For the surface roughness analysis, the 0.5 ​× ​0.5 ​μm^2^ image (1 ​Hz) was used to analyse the surface roughness of any given condition. The Peak-to-Valley roughness was calculated using the JPK DP software after image levelling to remove variations or tilts in the background.

The estimation of the coating thickness was performed according to our previous work [26]. Briefly, a thin scratch was manually applied with a sharp blade into the coating, to expose the underlying glass substrate. The surface was then viewed under a microscope to identify the scratch, and the area across the cut was scanned by AFM. The thickness of the polymer coating was estimated by profilometry at the boundary of the scratched and unscratched area, n ​= ​6 (minimum).

### 2.6 BMP2 enzyme-linked immunosorbent assay (ELISA)

After ECM absorption and subsequent GF absorption, the GF supernatant from each substrate was collected. The concentration of BMP2 in the stock solution and supernatant from each substrate was determined by the BMP2 Enzyme-linked immunosorbent assay (ELISA) kit (R&D System, #DY355-05) according to the manufacturer's protocol. In general, high-binding plates were coated by the capture antibody overnight. Then, blocking buffer, samples (both test samples and standard samples) and detection antibodies were subsequently added to the plate one by one and between each step, the plates were washed with 0.05% (v/v) Tween-20/PBS for 5 ​× ​5 ​min. After that, substrate solution was added and incubated for 20 ​min and then stop solution was added to stop the reaction. The ELISA plate was read using a plate reader at the wavelength of 450 ​nm and 570 ​nm. The absorbed BMP2 mass density was calculated by the following equation.

Equation 1 The equation for absorbed BMP2 density calculation.

ΔConcentration: The subtraction between the BMP2 concentration of the stock solution and the BMP2 concentration of the collected supernatant.

$$Absorbed\;BMP2\;density\;\left(ng/cm^{2}\right)=\frac{\Delta Concentration\;\left(ng/\mu l\right)\times \;Coated\;Volume\left(\mu l\right)}{well\;area\;\left(cm^{2}\right)}$$

### 2.7 P5F3 ELISA

P5F3 antibody was utilised to measure the availability of heparin II binding domains of FN [14]. After ECM absorption, samples were blocked with 1% bovine serum albumin (BSA)/PBS for 30 ​min at room temperature. Then, samples were incubated with primary antibody against the P5F3 domain (FN Heparin-binding domain, Santa Cruz Biotechnology, #sc-18827, 1:2000) for 1 ​h, followed by incubation with secondary antibody (goat-*anti*-mouse-HRP, ThermoFisher Scientific, #62–6520, 1:10,000) for 1 ​h. After that, the substrate solution (R&D System, #DY999) was added and incubated for 20 ​min. The reaction was stopped by adding the stop solution (R&D System, #DY994), and the ELISA plate was read using a plate reader at the wavelength of 450 ​nm.

### 2.8 Primary cell isolation and cell culture

Human MSCs were isolated from the bone marrow aspirates of patients undergoing joint replacement surgery. The permission to use the residual tissues was given by the Greater Glasgow and Clyde NHS Biorepository. Primary MSCs were maintained by Dulbecco's Modified Eagle Medium (DMEM, Sigma-Aldrich) supplemented with 10% Fetal Bovine Serum (FBS, Sigma-Aldrich), 1% sodium pyruvate (11 ​mg ​ml^−1^, Sigma-Aldrich), 1% Gibco non-essential amino acids (NEAA, ThermoFisher Scientific) and 2% antibiotics (6.74 U ​mL^−1^ penicillin-streptomycin and 0.2 ​μg ​ml^−1^ fungizone, Sigma-Aldrich). For experiments, MSCs (3000 ​cells cm^−2^) at the 2nd – 4th passage were seeded onto the materials, and the medium was changed twice a week. The experiments were repeated 3 times with MSCs from 3 patient donors. For the osteogenesis medium in the positive control group, the DMEM basal media was supplied with 100 ​nM Dexamethasone, 200 ​μM Ascorbic acid, and 10 ​mM β-glycerophosphate.

### 2.9 In-cell western (ICW)

After cell culture, the samples were fixed with 4% formaldehyde for 15 ​min at 37 ​°C and then permeabilised with 0.5% Triton-X/PBS, followed by blocking with 1% BSA/PBS for 1.5 ​h at room temperature. Samples were then incubated with primary antibody (pSMAD, #13820S, Cell Signalling Technology, 1:400) and then secondary antibody IRDye 800CW (LI-COR, #926–32211, 1:800) and CellTag 700 Stain (LI-COR, #926–41090, 1:500). After each incubation, samples were washed with 0.1% Tween-20/PBS 3 times for 5 ​min. The plates were dried in a fume hood, and then infrared signal readings were taken using an Odyssey infrared imaging system. Analysis was carried out by normalising the fluorescence units associated with the abundance of the protein of interest to the fluorescence units (in 800 ​nm channel) associated with the CellTag fluorescence units (in 700 ​nm channel).

### 2.10 Immunostaining

After cell culture, the samples were fixed with 4% formaldehyde for 15 ​min at 37 ​°C and then permeabilised by treating with 0.5% Triton-X/PBS, followed by blocking with 1% BSA/PBS for 5 ​min at 4 ​°C. Samples were incubated with primary antibody overnight at 4 ​°C (Phalloidin, #A12379, ThermoFisher Scientific, 1:200; Vinculin, #V9264, Sigma-Aldrich, 1:400; OPN, #sc-21742, Santa Cruz Biotechnology, 1:50; OCN, #sc-73464, Santa Cruz Biotechnology, 1:50) and then secondary antibody Texas red conjugated Goat anti-mouse secondary antibody for 1 ​h at room temperature. After each incubation, samples were washed with 0.1% Tween-20/PBS 3 times for 5 ​min. Samples were mounted using the mounting medium containing 4’, 6-diamidino-2- phenylindole (DAPI), before imaging in fluorescence microscopy. The images are acquired by EVOS M700 microscope (ThermoFisher Scientific). Images were processed and the integrated densities were determined by ImageJ.

Vinculin images were used for focal adhesion (FA) analysis according to the description by Horzum et al. [29]. Images were analyzed with a threshold area of 0.5 ​μm^2^ and 0–0.99 circularity. The binarized images of the FAs were measured and several parameters were used for FA analysis, including FA number per cell, the FA length and FA area.

### 2.11 Super-resolution microscopy

After the aforementioned immunostaining procedures, samples were ready for super-resolution microscopy. Structured Illumination microscopy (SR-SIM) was performed using a Zeiss Elyra PS.1 super-resolution microscope (Carl Zeiss, Germany). A plan-Apochromat 63x/1.4 Oil lens was used, and Z-steps of 0.2 ​μm were acquired (total thickness between 5 and 7 ​μm) in five rotations using the ZEN Black Edition Imaging software.

### 2.12 Von Kossa staining

After 28 days of culture, the samples were fixed with 4% formaldehyde for 15 ​min at 37 ​°C. Then 5% silver nitrate solution was added to the samples and the samples were kept under UV light for 30 ​min. After washing in deionised water, the samples were incubated with 5% sodium thiosulphate for 10 ​min at room temperature. After re-washing in deionised water, the samples were incubated by nuclear fast red for 10 ​min. The samples were washed with deionised water and washed with 70% ethanol for the final wash. After drying in the fume hood overnight, samples were ready for image acquisition. Light microscopy images were obtained using the EVOS M700 microscope (ThermoFisher Scientific). Integrated density measurements were obtained by using the LiCor Odyssey Sa system in the brightness setting.

### 2.13 Statistical analysis

Data were presented as means ​± ​standard deviation or means ​± ​standard error of the mean. Kruskal-Wallis test or Ordinary two-way ANOVA with Tukey's test was applied for multi-group comparison were applied for statistic analysis using GraphPad Prism 8TM software. The methods were stated as per figure legends.

## 3 Results

### 3.1 Chemical characterization of PEA substrates

**Fig. 2:**
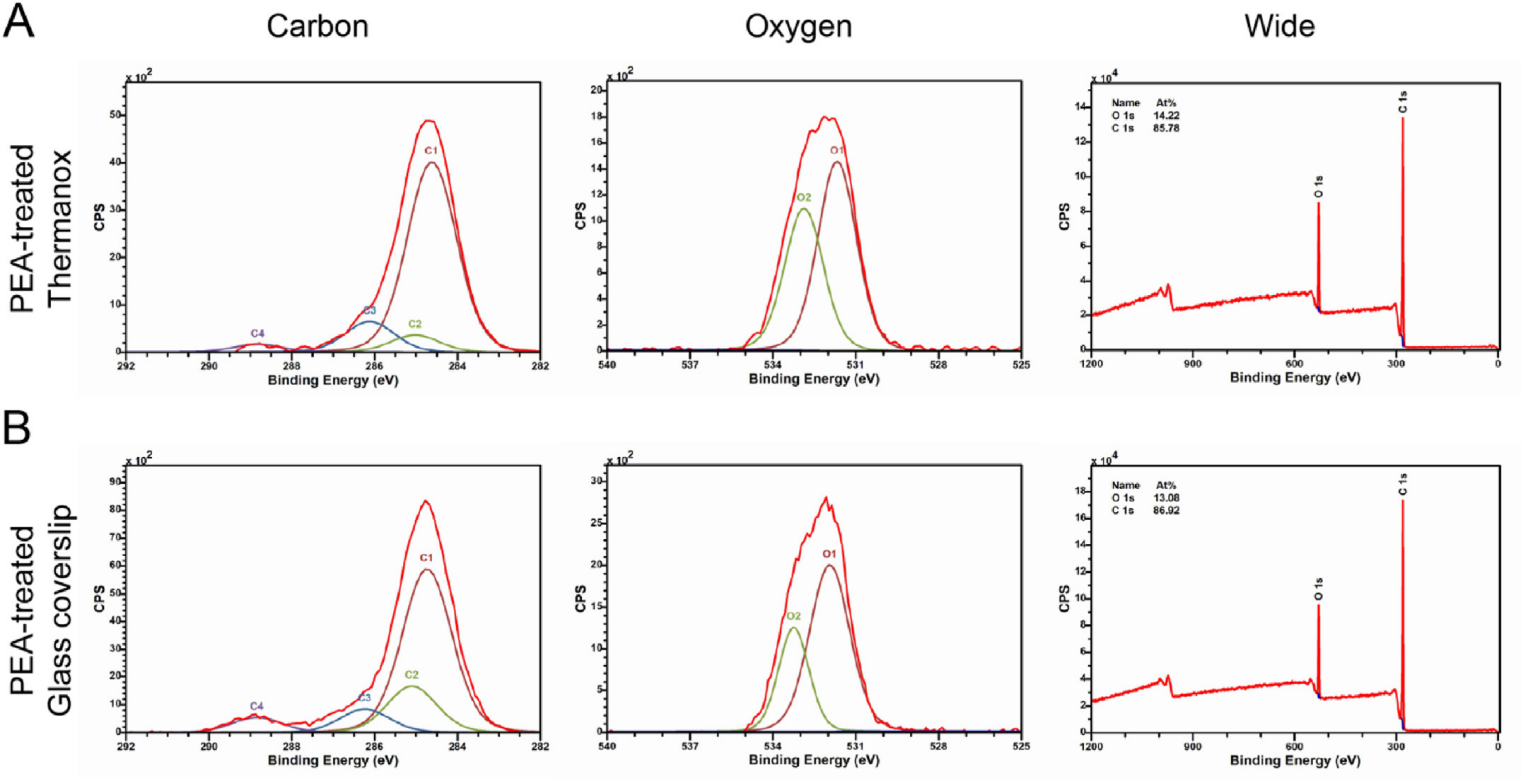
Chemical characterization of PEA treated surfaces. After plasma polymerization, the chemical signature of PEA was confirmed on both Thermanox and glass coverslips [13,26]. (A) and (B), the chemical composition of the surface measured by XPS analysis. The spectra from left to right were carbon, oxygen and wide scan respectively for PEA-treated Thermonox (A) and glass coverslips (B). In the carbon spectra, the presence of a large peak corresponding to C–C bonds found at 284.9 ​eV (C1) was used as a reference and used to identify peaks at 285.1 ​eV (C2), 286.2 ​eV (C3), 288.5 ​eV (C4) which correspond to the C–C–C bonds, C–O bonds, and C=O bonds respectively. In the Oxygen spectra, the peaks corresponding to the C=O and C–O–C bonds were found at 531.5 ​eV (O1) and 533.2 ​eV (O2) respectively. The red line corresponds to the overall fitting peak, and the lines with other colours correspond to the individual deconvoluted peaks representing the different functional groups. (For interpretation of the references to colour in this figure legend, the reader is referred to the Web version of this article.)

XPS analysis was carried out to determine the chemical composition and binding confirmation of the top 10 ​nm of the plasma polymerised substrates. The chemical structure of PEA was shown in Fig. 1B. In general, after plasma treatment, the chemical signature of PEA was confirmed on both glass and Thermanox tissue culture plastic coverslips (Fig. 2A and B). This was identified via reference to the literature [30] and previous PEA-related surface modification studies [14,31], as the PEA peaks were identical to those found in other plasma PEA studies [13,26]. The relative positions relating to the chemical motif of PEA could be identified; mainly the presence of a large peak corresponding to C–C bonds found at 284.9 ​eV (C1) as a reference and the peaks at 285.1 ​eV (C2), 286.2 ​eV (C3), 288.5 ​eV (C4) which correspond to the C–C–C bonds, C–O bonds, and C=O bonds respectively (Fig. 2A and B). As previously noted, the binding ratios between these 3 main peaks are slightly altered from pure PEA, due to plasma-induced monomer fragmentation [13,26], as plasma polymerization leads to a partial loss of functional groups and crosslinking. In the oxygen spectra, the peaks corresponding to the C=O and C–O–C bonds were found at 531.5 ​eV (O1) and 533.2 ​eV (O2) respectively (Fig. 2A and B). Several peaks were observed between 288 and 296 ​eV on untreated Thermanox coverslips within the carbon spectra which may correspond to the C=C bond in aromatic rings, whereas these carbon binding peaks, as well as nitrogen peaks in wide spectra, were not observed on scans after PEA treatment demonstrating successful coating (Fig. S1A, Supporting Information). Additionally, the non-treated glass coverslips possess relatively high percentages of silicon, as calculated from the wide scan (Fig. S1B, Supporting Information). However, post plasma polymerization, there was no silicon detected on the PEA-treated substrates, which only presented the elements carbon and oxygen on its surface. These results show that plasma polymerization can adequately cover the surfaces of both Thermanox and glass coverslips in PEA, with a thickness of 166 ​± ​18 ​nm (measured by AFM scratch test).

To sterilize and disinfect the biomaterials for biological application, UV irradiation is commonly utilised [32]. However, this can affect the surface chemical composition and thus limit their *in vitro* and *in vivo* use [32]. Thus, the static WCA measurement was carried out to determine if the PEA-treated coverslips were significantly altered by UV exposure. The WCA of PEA-treated coverslips before and after UV exposure were 77.12° ​± ​4.30 and 75.78° ​± ​4.11 respectively, with no statistical difference (Fig. S2, Supporting Information). This indicates that the UV treatment does not alter the PEA coating and that the sterilized coverslips can be further used for cell culture.

### 3.2 ECM conformation on PEA substrate

**Fig. 3:**
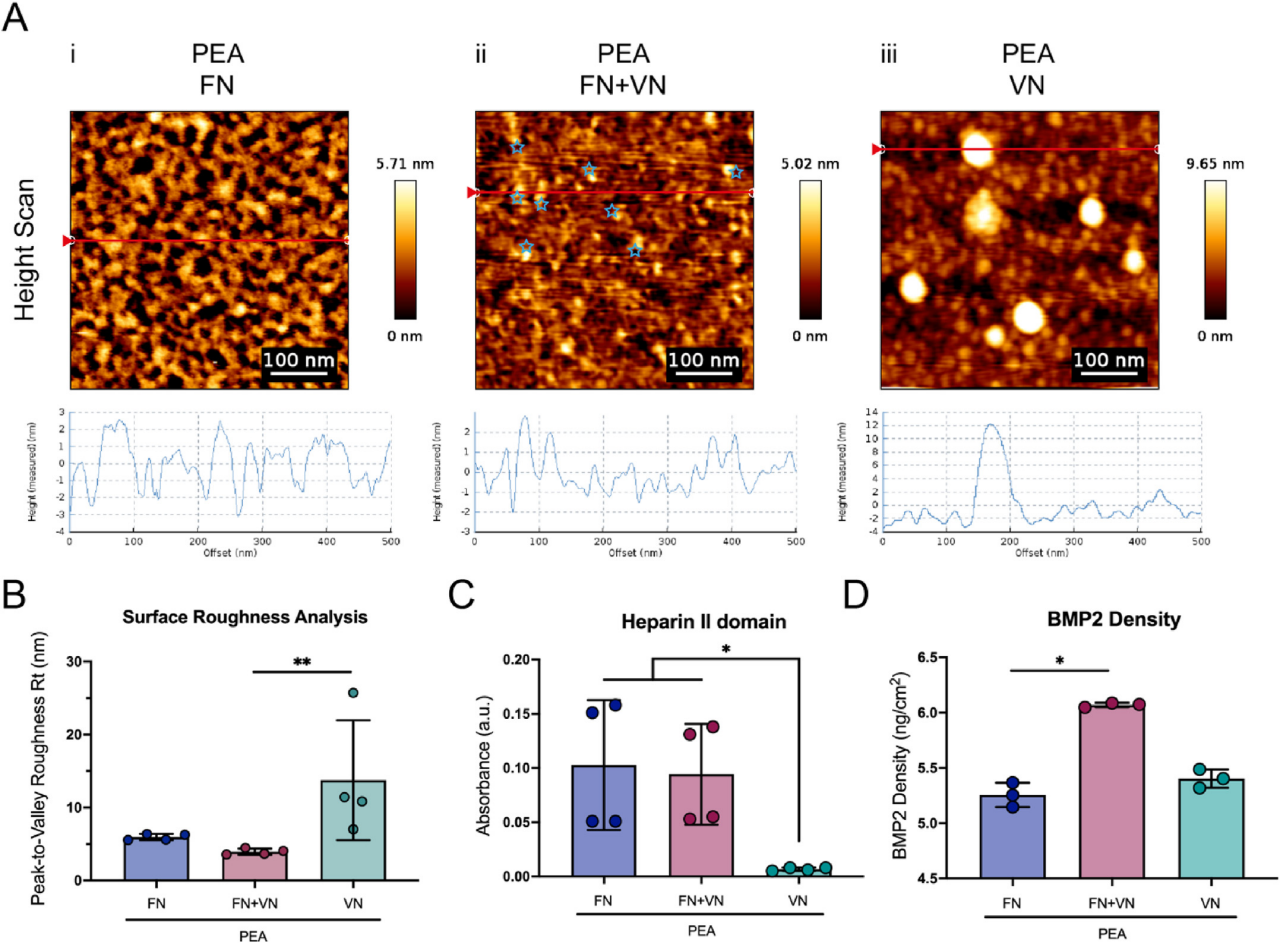
Physicochemical characterization of ECM and BMP-2 adsorption on FN/FN ​+ ​VN/VN coated PEA surfaces. (A), AFM height scan and phase images from FN (i), FN ​+ ​VN (ii), and VN (iii) PEA coated surfaces. The measured height corresponding to the cross-section (red line) was shown underneath each image. The FN on the PEA surface formed fibrillar networks, and VN on PEA remained in globular conformation/multimeric aggregates. Once co-coated with FN, VN was observed within the FN network (blue star). (B), Average peak-to-valley roughness analysis. Compared to the VN-only coating, the FN ​+ ​VN coating surface exhibited lower Peak-to-Valley roughness, indicating that the VN can incorporate into the FN fibrillar network. (C), The availability of the heparin II GF binding domain of FN was quantified by ELISA. The availability of this domain on the FN and FN ​+ ​VN surface was similar, but both are significantly more than on the VN alone surface. (D), The surface density of BMP2 on different ECM-coated PEA surfaces was quantified by ELISA. Significantly increased levels of BMP2 were detected on FN ​+ ​VN surfaces compared to FN only and VN only surfaces. The bar plot shows mean values and standard deviation. Each point represents one individual sample. n ​= ​3 or 4. Kruskal-Wallis test was applied for multiple comparisons. ∗p ​< ​0.05; ∗∗p ​< ​0.01. (For interpretation of the references to colour in this figure legend, the reader is referred to the Web version of this article.)

AFM was performed to determine the nanostructures formed by the absorbed proteins on the PEA surfaces. Plasma polymerised PEA surfaces without ECM coating were observed by AFM to be flat enough to allow visualisation in protein conformational studies as the low RMS roughness is unlikely to mask or hide any protein-related structures (Fig. S3, Supporting Information) [31]. It was seen that PEA coatings were able to drive fibrillogenesis of absorbed FN into fibrillar nanonetworks (Fig. 3A–i; Figs. S4–i, Supporting Information), resembling the fibrillar in-vivo FN organization [13,14]. In contrast, VN alone formed large, globular, aggregations of between 20 ​nm and 50 ​nm in height when absorbed onto PEA (Fig. 3A–iii; Fig. S4-iii, Supporting Information). The size of one single VN molecular is around 3–11 ​nm [24], suggesting the aggregates detected are multimers of VN molecules. When proteins (FN ​+ ​VN) were co-adsorbed, the large globular conformation of VN was not present on PEA surfaces, and, instead, FN nanonetworks were observed to present additional peaks which can be attributed to VN (Fig. 3A–ii (blue star); Fig. S4-ii, Supporting Information), as they were not present on PEA ​+ ​FN scans alone. Further, peak-to-valley roughness on the PEA ​+ ​FN ​+ ​VN surface was measured to significantly decrease compared to the PEA ​+ ​VN surface, with PEA ​+ ​FN ​+ ​VN instead presenting peaks within the range of a single VN molecule (Fig. 3B). The VN was observed within the FN fibrillar structure, allowing us to postulate that VN could likely be incorporated into the PEA-driven FN fibrillar networks.

### 3.3 The availability of functional domains and BMP2 absorption

The heparin II domain of FN is less accessible when the molecule is in its globular conformation. However, it is exposed once FN is in the fibrillar network [33]. The availability of heparin II binding domains was measured by ELISA using the antibody P5F3, which recognises the heparin II domain of FN [14,34]. As expected, there was more heparin II exposed on FN and FN ​+ ​VN coated surfaces compared to VN only (Fig. 3C). There was no significant difference between FN-only networks and the FN ​+ ​VN networks, indicating that the addition of VN has no positive or negative effect on FN heparin II domain exposure. Only a background signal was observed on PEA ​+ ​VN, indicating the antibody is specific to the heparin-II domain of FN. Given that there is no commercial antibody specific to the heparin-II domain of VN, the availability of the heparin-II domain of VN was not evaluated. BMP2 is known to bind to FN and VN [3]. To quantify BMP-2 adsorption on the coated substrates, a BMP2 ELISA was performed. Interestingly, compared to FN only, there was a significant increase in BMP2 adsorbed onto the FN ​+ ​VN surface, suggesting that the FN ​+ ​VN surface could increase the physiological, solid-state, presentation of BMP2 (Fig. 3D). This may be due to the ability of VN in improving fibril formation [23], thus exposing more GF binding regions. Notably, previous work has demonstrated that 90% of the adsorbed BMP2 remains on the surface of FN networks after 14 days [13], indicating such ECM-GF bonding is stable and thus retains GFs within the ECM network.

### 3.4 MSC adhesion on ECM-modified substrates

**Fig. 4:**
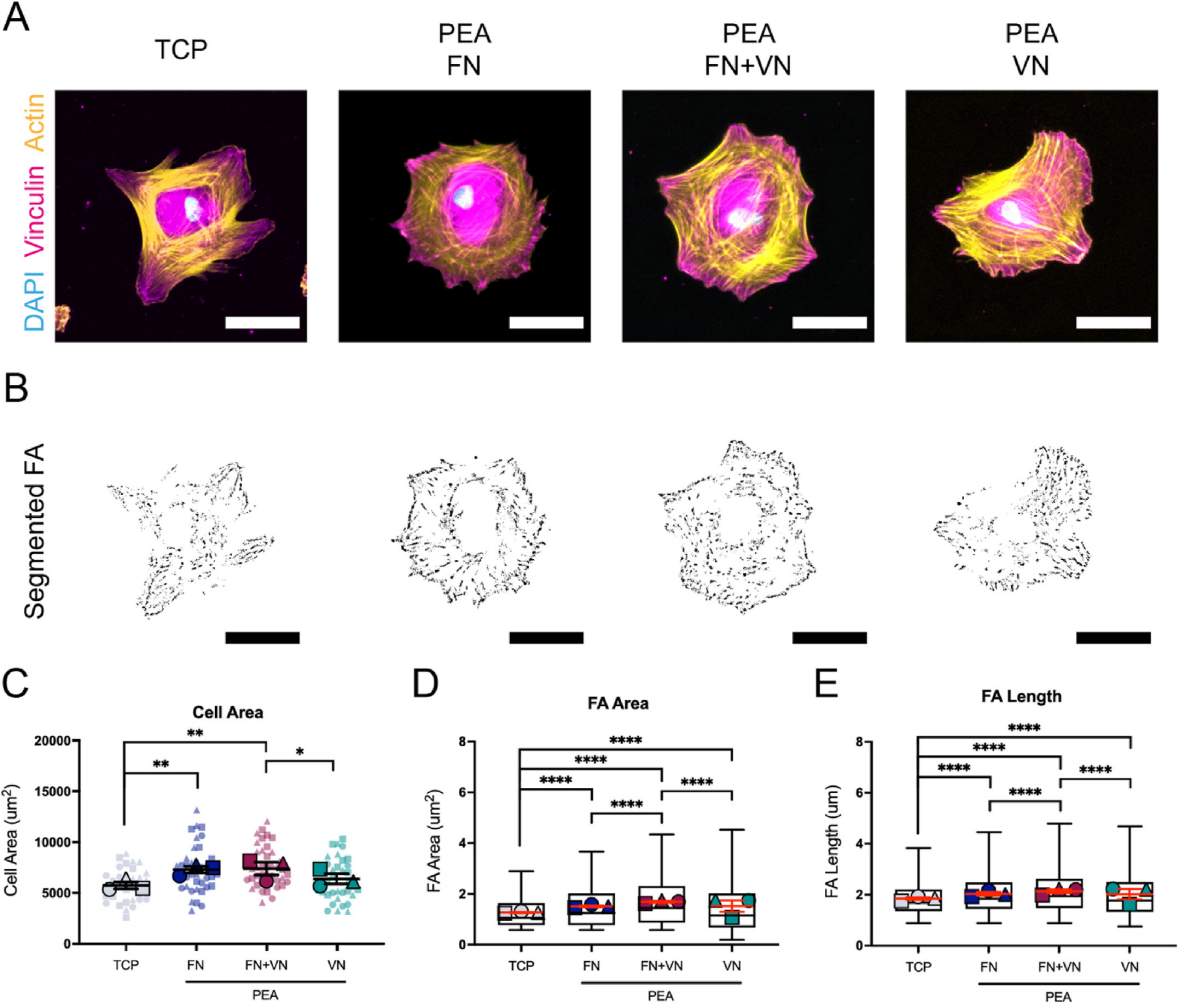
MSC spreading and adhesion on different ECM-modified substrates. MSCs were seeded onto different ECM-modified substrates for 3 ​h, followed by actin and vinculin staining. Cells on untreated TCP surfaces were set as a control. (A), Representative images of actin and vinculin staining. Actin (yellow), vinculin (purple), and DAPI (blue). The scale bar is 50 ​μm. (B), Representative images of thresholded binary images were obtained from analysis of the vinculin staining, highlighting the presence of mature exterior focal adhesions. The scale bar is 50 ​μm. (C), Cell area analysis based on the cellular actin staining images. (D), Average focal adhesion area analysis based on the cellular vinculin staining images. (E), Average focal adhesion length analysis based on the cellular vinculin staining images. Each shape represents a different donor, each small shape represents each cell measured, and each large shape represents the mean from each donor. The scatter plot shows mean values and standard deviation; box plots show the median and min/max, and the red lines in box plots represent the mean values and standard error of the mean. MSCs from 3 donors and n ​≥ ​10 ​cells/donor. Ordinary two-way ANOVA with Tukey's test for multiple comparisons. ∗p ​< ​0.05, ∗∗p ​< ​0.01, ∗∗∗p ​< ​0.001, ∗∗∗∗p ​< ​0.0001. (For interpretation of the references to colour in this figure legend, the reader is referred to the Web version of this article.)

To evaluate MSC adhesion onto different ECM coated substrates, cell morphology and cell adhesion were analyzed. Three hours after seeding, cells were stained for phalloidin and vinculin to analyse initial adhesion responses. In general, all coatings enhanced cell spreading compared to TCP control, with cells on FN and FN ​+ ​VN surfaces being the most spread (Fig. 4C). However, no significant difference was observed between FN only and FN ​+ ​VN groups (Fig. 4C; Fig. S5A, Supporting Information). The MSCs on FN and FN ​+ ​VN surfaces with significantly larger cell areas were also more rounded than those on the TCP control surface (Fig. 4C). Compared to VN alone, MSCs on FN ​+ ​VN exhibited significantly increased spreading, suggesting that the presence of FN could enhance VN-mediated cell spreading (Fig. 4C). Vinculin is a component of the focal adhesion (FA) complex. It is thought to stabilise talin unfolding, thus connecting the individual integrins with the actin cytoskeleton [35]. Therefore, as it is expressed throughout adhesions, measurement of the length of vinculin patterns correlates to the number of clustered integrins at each FA site. The ECM network surfaces demonstrated that, compared to the TCP control, MSCs developed larger and longer FAs with FN, VN/FN and VN coatings, indicating ECM protein coating enhances FA formation (Fig. 4D and E). Further, MSCs on FN ​+ ​VN surfaces developed larger (1.687 ​± ​0.972 ​μm^2^) and longer (2.140 ​± ​0.849 ​μm) FAs than the cells on FN alone (FA area 1.501 ​± ​0.809 ​μm^2^; FA length 2.029 ​± ​0.760 ​μm) or VN alone (FA area 1.472 ​± ​0.986 ​μm^2^; FA length 1.984 ​± ​0.870 ​μm) (Fig. 4D and E). While the overall differences were subtle, the data indicates that FN ​+ ​VN coatings could induce significantly increased cell adhesion, perhaps illustrating the importance of ECM heterogeneity.

**Fig. 5:**
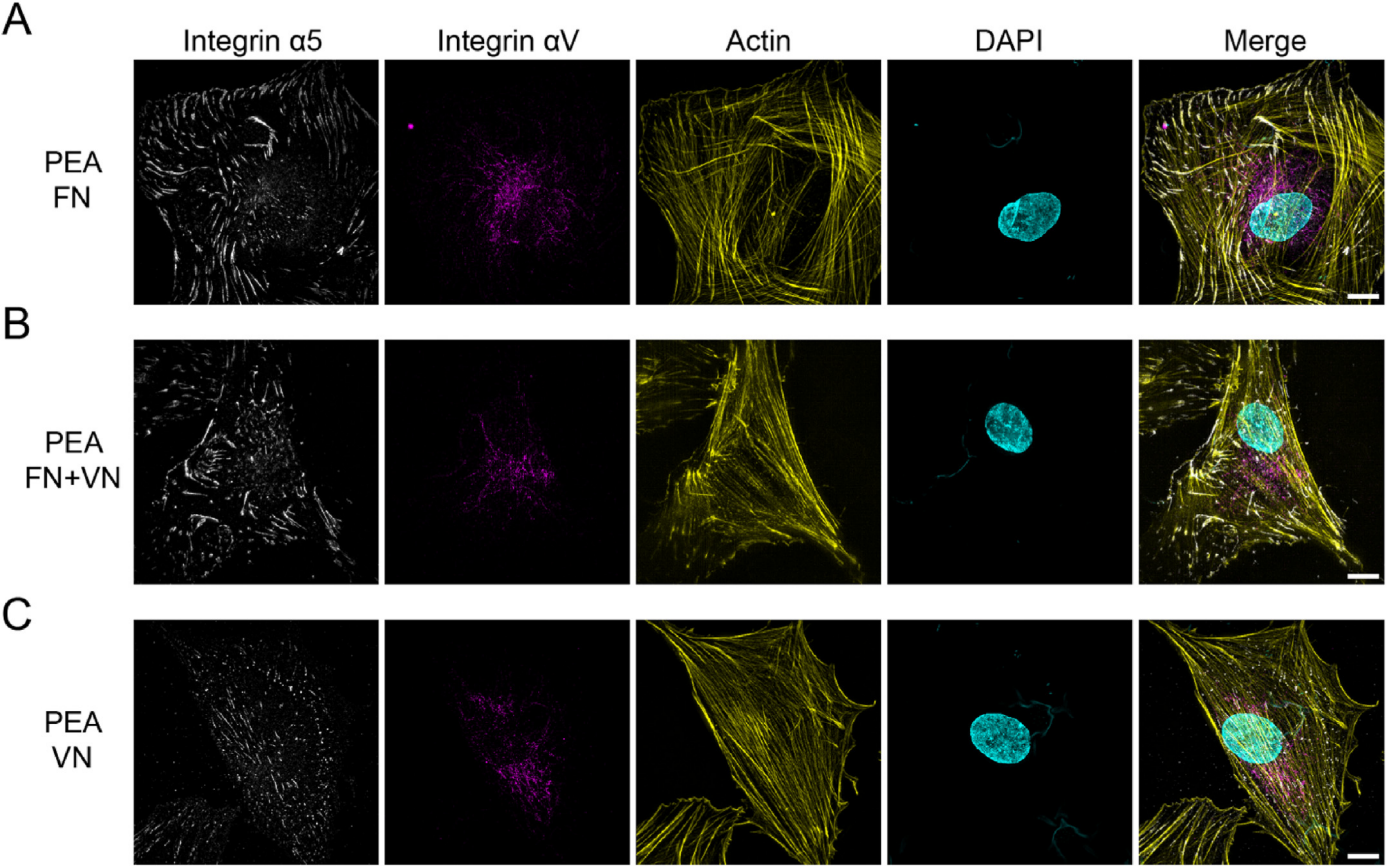
Super-resolution images of integrin α5 and αV on FN/FN ​+ ​VN/VN substrates. MSCs were seeded onto different substrates for 3 ​h, followed by integrin α5 (white), integrin αV (purple), actin (yellow) and DAPI (cyan) staining. (A), PEA ​+ ​FN substrate; (B), PEA ​+ ​FN ​+ ​VN substrate; (C), PEA ​+ ​VN substrate. Fibrillar α5 integrins could be observed within the MSCs on the FN and FN ​+ ​VN substrates, while α5 integrins kept dot-like within the MSCs on VN substrates. No significant difference was observed in αV integrins distribution on these substrates. The scale bar is 10 ​μm. (For interpretation of the references to colour in this figure legend, the reader is referred to the Web version of this article.)

Different integrins are known to couple to different ECM proteins. Integrin α5 is known to have high specificity for binding FN, while αV is found to bind to both FN and VN [5,36,37]. Super-resolution microscopy was performed to determine the integrin type in the FAs on each ECM-coated surface. As expected, fibrillar α5 integrins were observed at the end of actin filaments for MSCs on FN and FN ​+ ​VN surfaces but appeared more diffuse/less fibrillar on VN only surface (Fig. 5, First lane). In terms of αV integrin distribution, most αV integrins were distributed in the perinuclear region rather than at the cell periphery for all conditions (Fig. 5, Second lane).

### 3.5 Immobilized BMP2 enhances cell adhesion

**Fig. 6:**
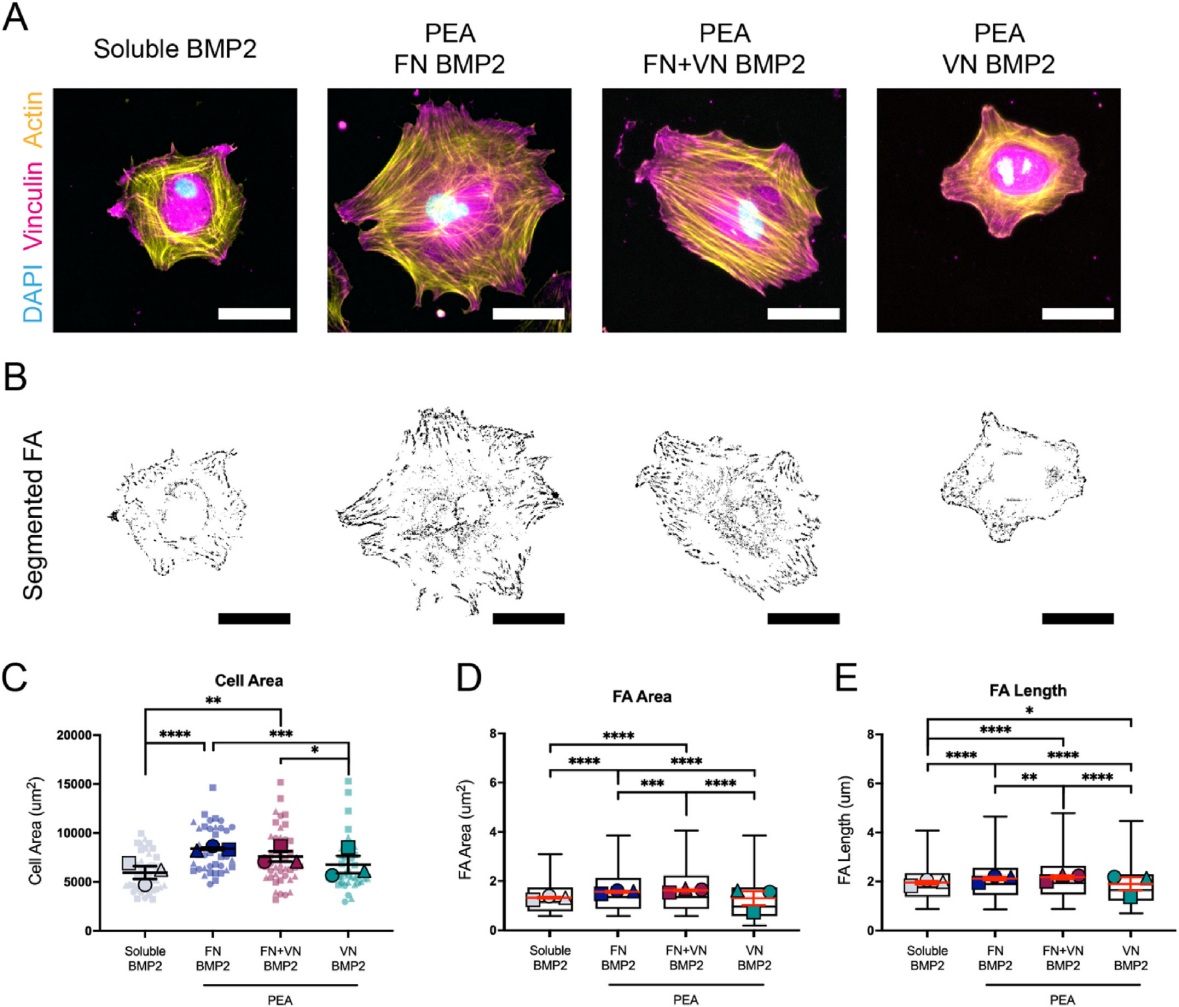
MSC spreading and adhesion on different ECM ​+ ​BMP2-modified substrates. MSCs were seeded onto different ECM ​+ ​BMP2 coated substrates for 3 ​h, followed by actin and vinculin staining. Cells treated with the soluble BMP2 were set as a control. Immobilized BMP2 significantly promoted MSCs spreading and enhanced FA assembly compared to soluble BMP2. MSCs on the FN ​+ ​VN ​+ ​BMP2 substrates displayed increased spreading and longer and larger FA than FN ​+ ​BMP2 and VN ​+ ​BMP2 substrates. (A), Representative images of actin and vinculin staining. Actin (yellow), vinculin (magenta), and DAPI (blue). The scale bar is 50 ​μm. (B), Representative images of thresholded binary images were obtained from analysis of the vinculin staining. The scale bar is 50 ​μm. (C), Cell area analysis. (D), Average focal adhesion area analysis. (E), Average focal adhesion length analysis. Each shape represents a different donor, each small shape represents each cell measured, and each large shape represents the mean from each donor. The scatter plot shows mean values and standard deviation; box plots show the median and min/max, and the red lines in box plots represent the mean values and standard error of the mean. MSCs from 3 donors and n ​≥ ​10 ​cells/donor. Ordinary two-way ANOVA with Tukey's test for multiple comparisons. ∗p ​< ​0.05, ∗∗p ​< ​0.01, ∗∗∗p ​< ​0.001, ∗∗∗∗p ​< ​0.0001. (For interpretation of the references to colour in this figure legend, the reader is referred to the Web version of this article.)

The synergistic crosstalk between GF receptors and integrin receptors can regulate actin organization, cell adhesion and cell differentiation [4,7]. By functionalizing BMP2 onto FN/FN ​+ ​VN/VN coated surfaces, the effects of matrix-bound BMP2 and ECM composition on cell adhesion were evaluated based on phalloidin and vinculin staining. Compared to soluble BMP2, immobilized BMP2 promoted MSC spreading and adhesion. MSCs had an increased surface area on all ECM ​+ ​BMP2 surfaces (Fig. 6C). However, MSCs on FN ​+ ​BMP2 and FN ​+ ​VN ​+ ​BMP2 had significantly increased surface areas than those on VN ​+ ​BMP2 (Fig. 6C). Interestingly, analysis of FAs revealed that FN ​+ ​VN ​+ ​BMP2 surface led to the development of larger (1.620 ​± ​0.920 ​μm^2^) and longer (2.150 ​± ​0.862 ​μm) FAs than the ones on the FN ​+ ​BMP2 (FA area 1.556 ​± ​0.865 ​μm^2^; FA length 2.095 ​± ​0.828 ​μm) and VN ​+ ​BMP2 (FA area 1.247 ​± ​0.884 ​μm^2^; FA length 1.841 ​± ​0.835 ​μm) substrates (Fig. 6D and E), even though the differences were subtle. These indicated the addition of GFs such as BMP-2, may further enhance cell adhesion, but only when the GF is presented in solid-state and sequestered by the ECM, rather than as soluble administration.

**Fig. 7:**
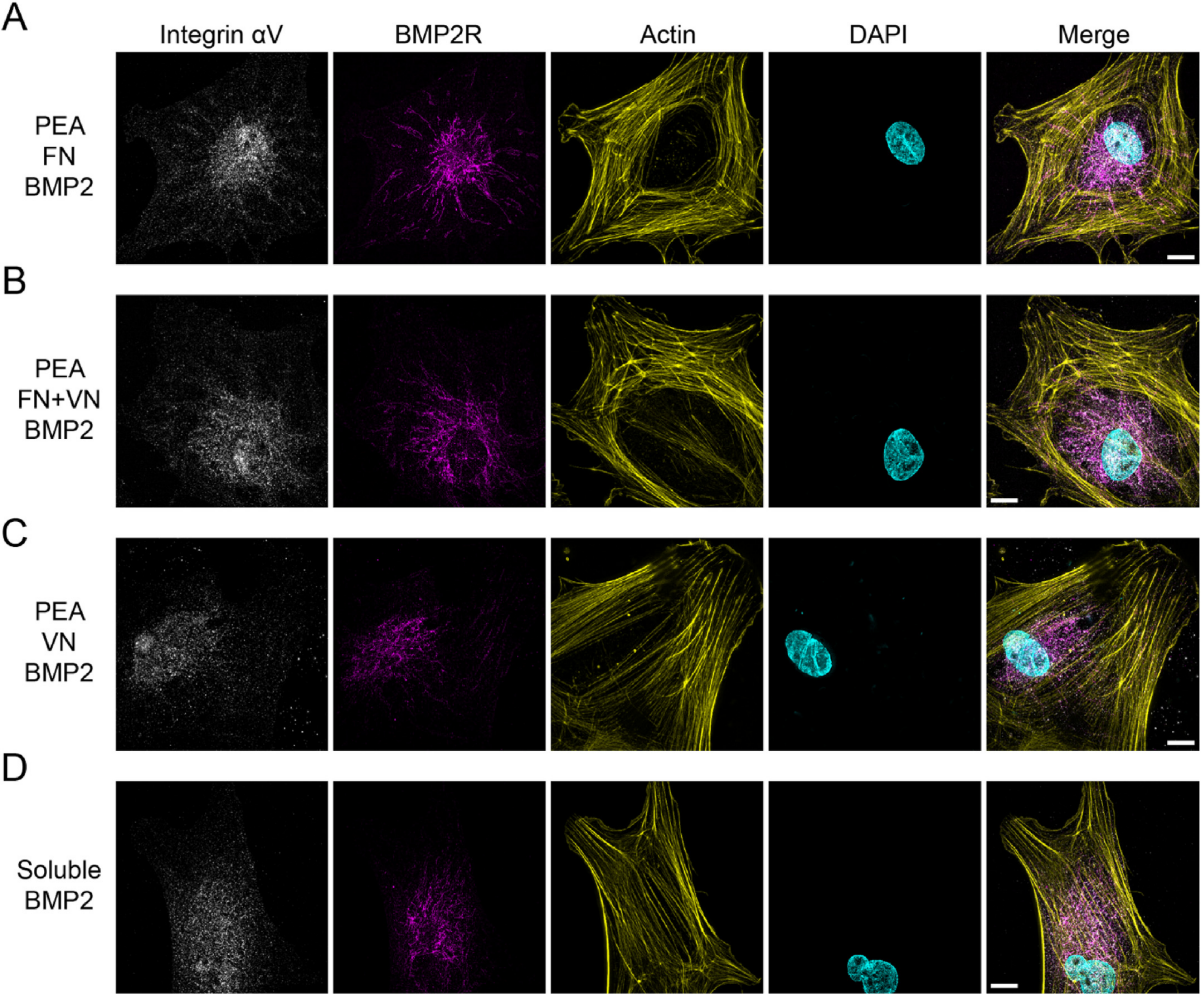
Super-resolution images of Integrin αV and BMP2R on FN/FN ​+ ​VN/VN ​+ ​BMP2 substrates. MSCs were seeded onto different substrates for 3 ​h, followed by integrin αV (white), BMP2R (magenta), actin (yellow) and DAPI (cyan) staining. MSCs treated with soluble BMP2 were set as a control. (A), PEA ​+ ​FN ​+ ​BMP2 substrate; (B), PEA ​+ ​FN ​+ ​VN ​+ ​BMP2 substrate; (C), PEA ​+ ​VN ​+ ​BMP2; (D), MSCs treated with soluble BMP2. BMP2R could be observed to be fibrillar and organized, extending from the centre to the edge of the cells within the MSCs on the FN ​+ ​BMP2 and FN ​+ ​VN ​+ ​BMP2 substrates, while MSCs on VN ​+ ​BMP2 and soluble BMP2 groups BMP2R remains concentrated in the perinuclear area. No significant difference was observed in αV integrin distribution on these substrates. The scale bar is 10 ​μm. (For interpretation of the references to colour in this figure legend, the reader is referred to the Web version of this article.)

Given the synergy of GF receptors and integrins, the morphological features of the BMP2 receptor (BMP2R) and integrin αV were characterised by super-resolution microscopy. When BMP2 was presented in solid-state, the BMP2 receptors were observed to take on a fibrillar appearance at the cell edge of MSCs on FN ​+ ​BMP2 and FN ​+ ​VN ​+ ​BMP2 substrates (Fig. 7A and B, Second lane). However, with MSCs seeded on VN ​+ ​BMP2 substrates and the MSCs treated with soluble BMP2, the BMP2R were mostly distributed in the perinuclear region, and were dot-like in appearance, rather than having an organized fibrillar morphology (Fig. 7C and D, Second lane). As for the αV integrin, there was no significant morphology difference in αV integrin on the different ECM surfaces (Fig. 7, First lane).

### 3.6 FN ​+ ​VN BMP2 surface enhances MSC osteogenesis

**Fig. 8:**
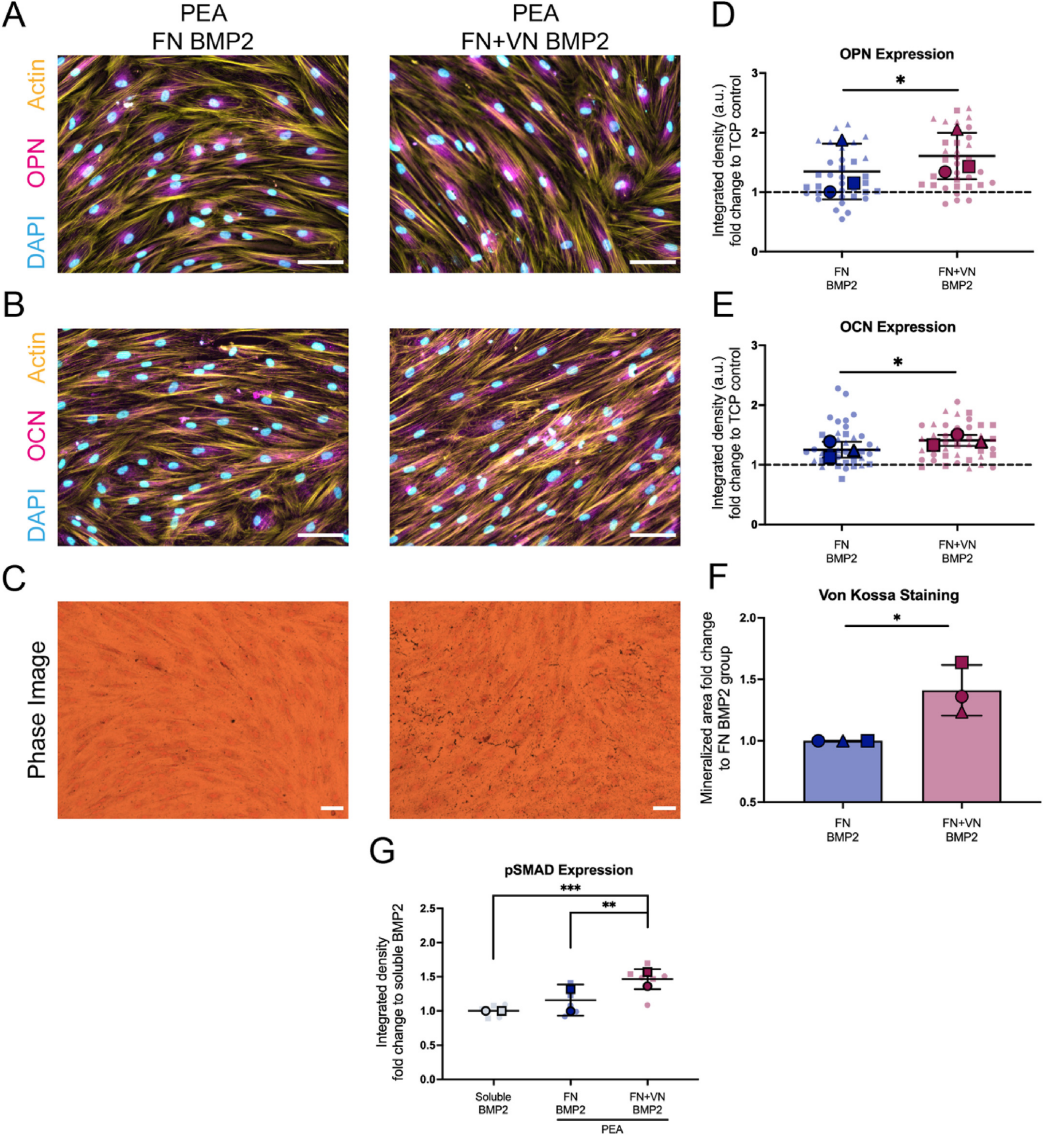
MSC osteogenic differentiation on different ECM ​+ ​BMP2 modified substrates. MSCs were cultured on different substrates for 28 days, followed by OPN and OCN staining, as well as von Kossa staining. FN ​+ ​VN ​+ ​BMP2 substates could significantly support MSC osteogenic differentiation, in terms of OPN and OCN expression as well as mineralization deposition via increased activation of the pSMAD 1/5/9 pathway. (A), Representative images of OPN staining from MSCs on FN/FN ​+ ​VN ​+ ​BMP2 substates. Scale bar 100 ​μm. (B), Representative images of OCN staining from MSCs on FN/FN ​+ ​VN ​+ ​BMP2 substates. The scale bar is 100 ​μm. (C), Representative images of von Kossa staining from MSCs on FN/FN ​+ ​VN ​+ ​BMP2 substates. Scale bar 100 ​μm. (D) and (E), Quantification of immunofluorescence images of OPN and OCN staining from MSCs on FN/FN ​+ ​VN ​+ ​BMP2 substates. MSCs cultured on TCP surfaces were used as a control. Fluorescence intensity fold change to the TCP group was analyzed. MSCs were isolated from 3 donors. (F), Quantification of mineralized area based on von Kossa staining from MSCs on FN/FN ​+ ​VN ​+ ​BMP2 substates. Mineralized area fold change to the FN BMP2 group was analyzed. MSCs were isolated from 3 donors. (G), Quantification of pSMAD ICW results. MSCs treated with soluble BMP2 were set up as a control. Fluorescence fold change to the soluble BMP2 group was analyzed. MSCs were isolated from 2 donors. Each shape represents a different donor, each small shape represents each cell measured or each technical replicate, and each large shape represents the mean from each donor. The scatter plot and the bar plot show mean values and standard deviation. Paired T-test was applied for two-group comparisons. Ordinary two-way ANOVA with Tukey's test was applied for multi-group comparison. ∗p ​< ​0.05, ∗∗p ​< ​0.01.

Given that FN ​+ ​VN surfaces adsorbed increased BMP2 (Fig. 3D), promoted cell adhesion (Fig. 6) and drove BMP2R as a fibrillar appearance (Fig. 7), we hypothesised that these substrates could enhance the osteogenic differentiation of MSCs. With this hypothesis, MSCs were cultured on ECM-coated surfaces for 28 days, and the expressions of osteoblastic markers osteopontin (OPN) and osteocalcin (OCN) were evaluated. During the culture, most MSCs on the VN ​+ ​BMP2 surface detached from the substrates at day ∼14, thus this condition was excluded from the analysis. MSCs grown in the osteogenic medium were used as a positive control, these expressed OPN and OCN, as well as supporting matrix mineralization, confirming the osteogenic potential of the cells (Figs. S6 and S7, Supporting Information). As expected, FN ​+ ​VN ​+ ​BMP2 substrates led to increased expression of OPN and OCN, when compared to FN ​+ ​BMP2 substrates (Fig. 8A, 7D and 8B, 7E). Further to this, matrix mineralization was confirmed by von Kossa staining. MSCs cultured on FN ​+ ​VN BMP2 surfaces showed a significantly higher level of mineralization compared to the FN ​+ ​BMP2 surface (Fig. 8C and F). Taken together, the FN ​+ ​VN ​+ ​BMP2 surface was able to significantly enhance the osteogenic differentiation of MSCs.

The SMAD signalling pathway is critical in BMP2-mediated MSC osteogenesis. Upon GF binding, SMADs 1, 5 and 9 can be phosphorylated by BMP2R, leading to their translocation to the nuclei to activate the osteogenic Runt-related transcription factor 2 (RUNX2) [38]. Thus, we sought to investigate the SMAD 1/5/9 activation. In-cell western (ICW) was performed to assess SMAD 1/5/9 phosphorylation in MSCs seeded on different substrates. Increased phosphorylated SMAD (pSMAD) was detected within cells on FN ​+ ​VN ​+ ​BMP2 when compared to cells on the FN ​+ ​BMP2 substrates and the ones treated by soluble BMP2 (Fig. 8G). These results suggested that BMP2-SMAD signalling was enhanced in MSCs by culture on the FN ​+ ​VN ​+ ​BMP2 surface.

## 4 Discussion

In this new report, we evaluate the effect of solid-state, ECM-based, presentation of FN and VN on MSC adhesion and MSC osteogenic differentiation. XPS analysis confirmed the successful polymerization of PEA onto TCP substrates (Fig. 2). The chemical signature of PEA polymerised substrates was similar to examples shown in previous work [13,26]. By AFM, VN was observed within the FN fibrillar network on the FN ​+ ​VN absorbed PEA surface, postulating that VN could be likely incorporated with the PEA-driven FN fibril (Fig. 3A–ii). This physiological-like network structure promoted MSC spreading and adhesion (Figs. 4 and 6; Fig. S5, Supporting Information). Then, BMP2 was adsorbed, leading to solid-state GF presentation to MSCs. Our results indicated that increasing the complexity of the ECM network from FN only to incorporate FN ​+ ​VN, led to enhanced BMP2-mediated osteogenesis of MSCs. We observed up-regulation of osteoblastic marker expression, OPN and OCN, as well as mineralization deposition (Fig. 8; Figs. S6 and S7, Supporting Information). Moreover, the increased phosphorylation of SMAD 1/5/9 signalling was observed on FN ​+ ​VN ​+ ​BMP-2 substrates, which may contribute to the enhanced osteogenesis.

The absorption, conformation and bioactivity of the ECM proteins are dependent on the surface chemical modification and surface hydrophobicity [39]. Via functionalization with different chemical groups (e.g. CH_3_, NH_2_, OH), self-assembled monolayers have been utilised to systematically study the absorption and conformation of FN and VN coated onto these surfaces [40,41]. Overall, both FN and VN adsorbed more onto hydrophobic surfaces, for example, functionalized with the CH_3_ groups [42,43]. VN absorbtion onto such a hydrophobic surface, however, resulted in the hiding of cell adhesion domains [40]. In our present study, the globular conformations of VN were confirmed on the hydrophobic PEA surface as well, theoretically hiding their integrin-binding domains and thus attenuating cell adhesion (Fig. 3, Fig. 4). Interestingly, compared to the VN alone, the addition of FN into the VN coating promoted cell adhesion, indicating that more cell-binding domains were exposed when FN and VN were co-adsorbed on the PEA surface. Thus, we can postulate that incorporation into the FN networks could enhance the exposure of the VN cell-binding domain. Due to the lower molecular weight of VN than FN, FN perhaps absorbs first, and VN absorbs to the PEA surface subsequently. The pre-adsorbed FN layer on a fully covered substrate lowers the hydrophobicity of the surface, perhaps allowing the conformational transition of VN to reveal RGD [40]. Moreover, as VN is known to interact with other ECM proteins via their heparin-binding domains [15,44], the pre-adsorbed FN layer can be postulated to provide further sites for VN interactions. Future studies should be carried out to clarify these hypotheses.

Integrins are generally believed to mediate cell-ECM interaction, subsequently stimulating the intracellular downstream signal pathways after initiating cell adhesion [5]. In our present study, we observed that α5 integrins can form fibrillar clusters in the MSCs adherent to the FN and FN ​+ ​VN surface (Fig. 5). In contrast, α5 integrins were observed to be dot-like in the MSCs adherent to the VN-only surface, suggesting that the VN is insufficient to induce α5 integrin clustering. These results again highlight that the α5 integrins canonically bind to FN, rather than VN [19,33]. The fibrillar α5 integrins are capable of activating RhoA activity in cells and driving the formation of contractile actin stress fibres [45,46]. Interestingly, in the context of cell area and FA development, we found that the mix of FN and VN on the PEA surface could promote cell adhesion better than FN alone (Fig. 4; Fig. S5, Supporting Information), indicating the important role of heterogeneous ECM composition in improving biomaterial-cell interaction.

Matrix-bound GFs allow for a robust synergy between the integrins and GF receptors [47]. In our present study, compared to the BMP2 in soluble format, BMP2 bound within the matrix can significantly induce cell spreading and FA development. The activated GF receptors within the matrix can act synergistically with integrin-related signals, amplifying both effects [47]. As a result, BMP2 presented in a matrix-bound form can strengthen early cell adhesion events, including adhesion and migration [[48], [49], [50]]. As the αvβ3 activation has been implicated in BMP2-dependent cell adhesion [49], αv integrins were stained and their assembly was analyzed. However, no significant difference in αv integrin appearance was observed among all the groups studied (Fig. 7). It is, therefore, likely that BMP2-induced cell adhesion on the FN ​+ ​VN surface is αvβ3-independent. Indeed, it has been shown that for surfaces presenting immobilized BMP2, αvβ3 integrin blocking either did not affect cell spreading [48]. It should be noted that the α5β1 integrins are involved in BMP2-mediated cell adhesion as well, and this is deserving of further investigation [48,51].

In our present study, compared to BMP2 delivered in a soluble manner, BMP2 presented in matrix-bound (FN and FN ​+ ​VN) format significantly activated the BMP2-dependent SMAD signalling pathway (Fig. 8G), in which SMADs 1, 5 and 9 can be phosphorylated and translocated to nuclei to activate osteogenesis-related transcriptional factors, such as RUNX2 [38]. These results emphasized again the concept that the matrix presentation of GFs can result in high efficiency, in terms of activation of biological functions, even at a very low dose [52,53]. Strikingly, our super-resolution images illustrated that matrix-bound BMP2, rather than the soluble BMP2 could induce BMP2 receptors to cluster with a fibrillar appearance (Fig. 7), even though the exact mechanism for this cluster is currently unknown. We speculated that similarly to the integrin clustering, BMP2 receptors can cluster as well. The cytosolic tyrosine kinase focal adhesion kinase (FAK) can bridge the cytoplasmic tails of growth factor receptors and integrins through its amino and carboxyl termini, respectively [54,55]. Once integrins cluster, GF receptors could be spontaneously clustered, leading to the formation of the integrin-growth-factor-receptors complex [47]. Again, further investigation is required to provide definitive evidence for this hypothesis.

It should be noted that although traditional culture methods that supplement various GFs into media have been valuable in our understanding of the effects of GFs on cell behaviours, these methods have disadvantages, such as high cost, supraphysiological dose administration, and off-target effects [4,7]; these disadvantages have been taken into clinic. For example, the high dose of BMP2 (1.5 ​mg/ml; INFUSE Bone Graft; Medtronic) was approved by the Food and Drug Administration for spinal fusion and the treatment of acute, open tibial shaft fractures [56]. However, in their clinical practice, various adverse events such as surgical site infection and heterotopic bone were reported [[57], [58], [59]]. Thus, new biomaterials require engineering to deliver BMP2 in a bioactive form at a low dose, so as to achieve higher osteogenic efficiency safely [1,51,60]. For example, protein engineering techniques, including the use of peptides that bind heparin and GFs [61], and the use of layer-by-layer technologies [62], are reported to be effective to deliver GFs from material surfaces. However, these approaches do not exploit the robust synergy between the integrins and GF receptors to enhance accelerated regeneration [52].

The PEA system allows for physiological, ultralow doses (ng) of GFs to be tethered within the matrix, leading to more precise and effective presentation to cells, followed by on-target guiding of cell differentiation. As a proof of concept, in our previous and present studies, BMP2, and VEGF, have been adsorbed within the ECM-modified PEA surfaces, with promising outcomes in the field of bone formation and vascularization both *in vitro* and *in vivo* [[12], [13], [14]]. Thus, PEA holds great potential for use in the field of tissue engineering, especially where delivery GFs is important to achieve cellular effects.

## 5 Conclusion

In conclusion, we have shown that PEA surface coatings are an optimal platform for cell-material interaction studies. Further to this, we demonstrate that combining FN and VN into fibrillar networks promotes MSC adhesion, efficiently presents of BMP2 to MSCs, and enhances BMP2-mediated MSC osteogenic differentiation. This work also demonstrates the use of the PEA system to recapitulate ECM *ex vivo* with controlled degrees of complexity. This is a useful tool for the investigation into cell adhesion dynamics and integrin-GF receptor crosstalk studies. This system may offer insights into therapeutically relevant tissue engineering applications that can be utilised *in the clinic*. Given that the PEA has already been used in veterinary trials with FN and BMP2, it is easily envisioned that systems with differing ECM compositions and complexities could be utilised for varied applications *in the clinic.*

## Declaration of competing interest

The authors declare that they have no known competing financial interests or personal relationships that could have appeared to influence the work reported in this paper.
