# Supplementary material for: Material-driven fibronectin and vitronectin assembly enhances BMP-2 presentation and osteogenesis
Source: Mater Today Bio. 2022 Jul 19;16:100367. doi: 10.1016/j.mtbio.2022.100367 (PMC9352550; doi:10.1016/j.mtbio.2022.100367)
Supplement: Multimedia component 1 [file mmc1.docx]

# Supporting Information


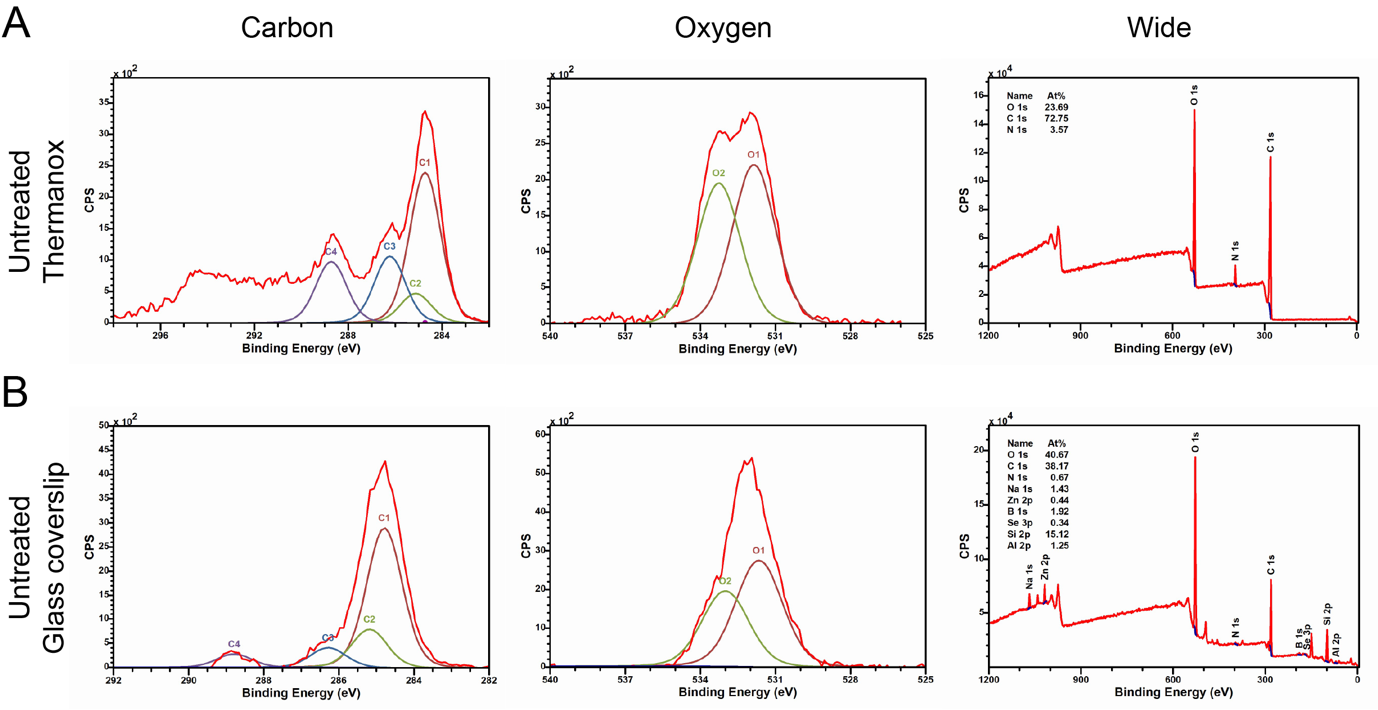


**Figure S1** Chemical composition of the PEA coating measured by XPS analysis. (A), Chemical composition of the untreated Thermanox coverslip surface; (B), Chemical composition of the untreated glass coverslip surface. The lanes from left to right were Carbon spectra, Oxygen spectra and Wide spectra, respectively. Several peaks were detected between 288 to 296 eV on untreated Thermanox coverslips within the C1s carbon spectra which may correspond to the C=C bond in aromatic rings. The non-treated glass coverslip possesses relatively high percentages of silicon, as calculated from the wide scan. There were not detected post plasma PEA polymerization. The red line corresponds to the overall fitting peak, and the lines with other colours correspond to the individual deconvoluted peaks, which represent the different functional groups.


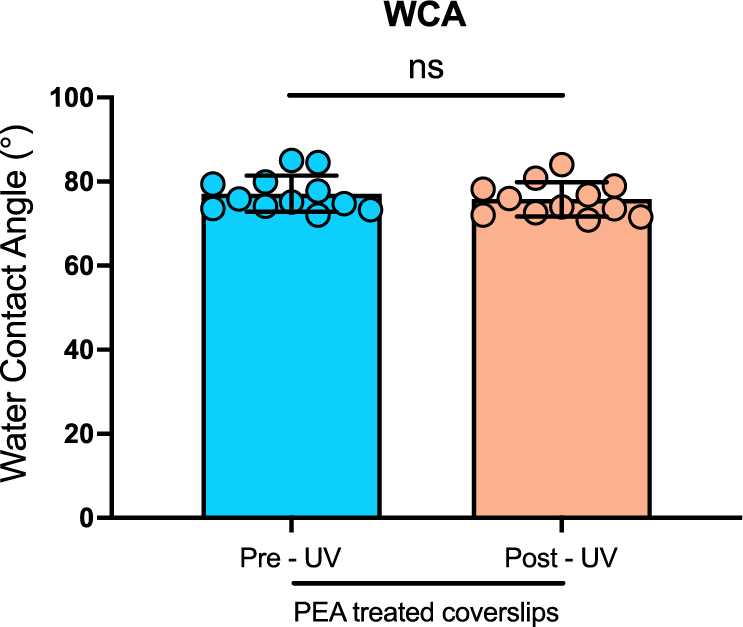


**Figure S2** WCA measurement of PEA-treated coverslips before and after UV exposure. WCA measurement was carried out to determine if the stability of PEA-treated coverslips was changed by UV exposure. No significant difference in the WCA of PEA-treated coverslips was observed before and after UV exposure. The bar plot shows mean values and standard deviation. Each point represents sample replicates, n=12. Mann-Whitney test was applied for the comparison. “ns”, “not significant”.


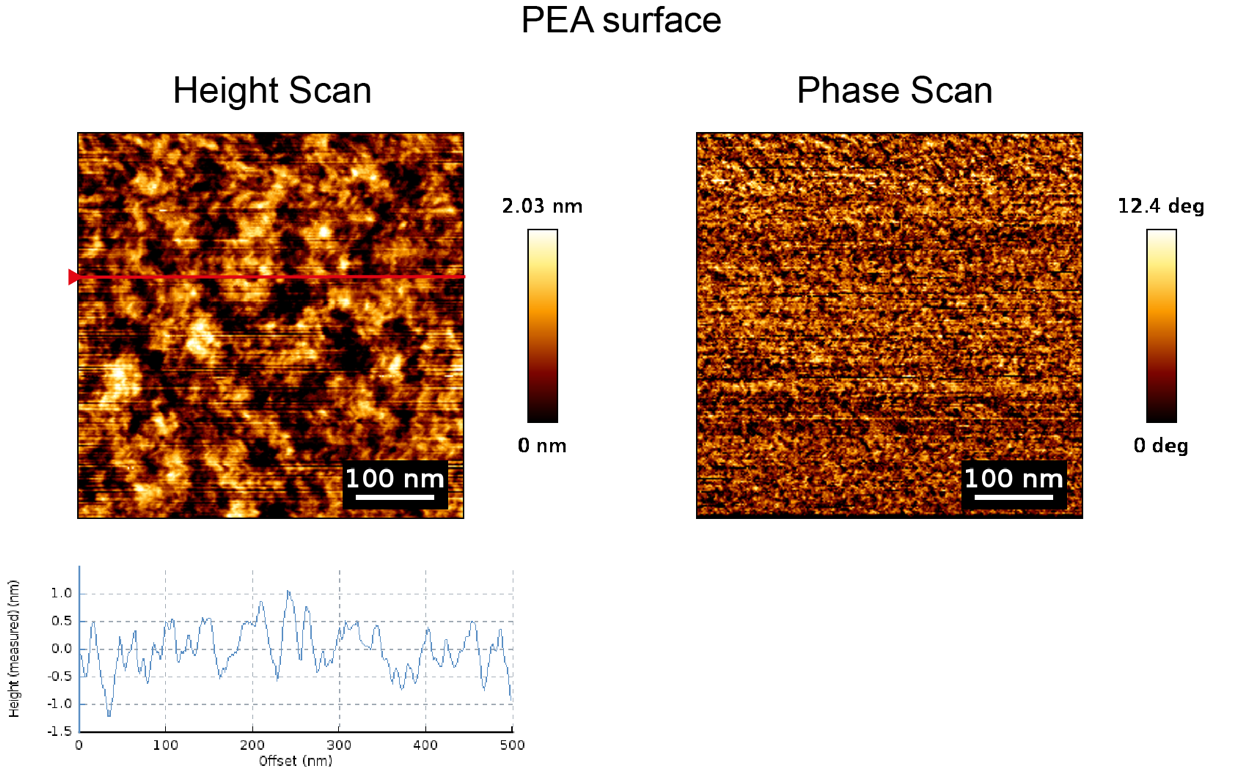


**Figure S3** The AFM images of PEA surface before protein absorbtion. Height Scan (Left), Phase Scan (Right). The measured height corresponding to the cross-section (red line) was shown underneath each image.


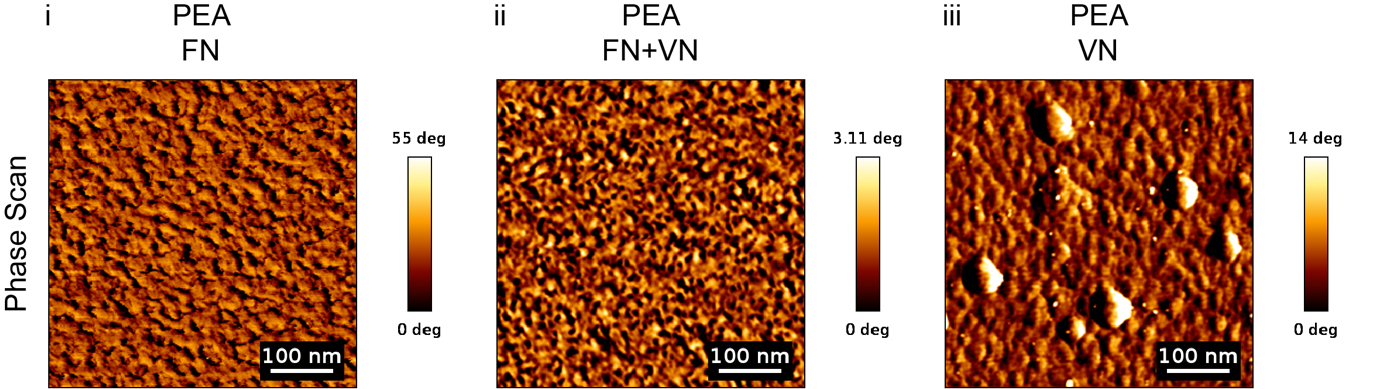


**Figure S4** The AFM Phase scan images from FN/FN+VN/VN coated PEA surfaces. PEA+FN (i); PEA+FN+VN (ii); PEA+VN (iii).


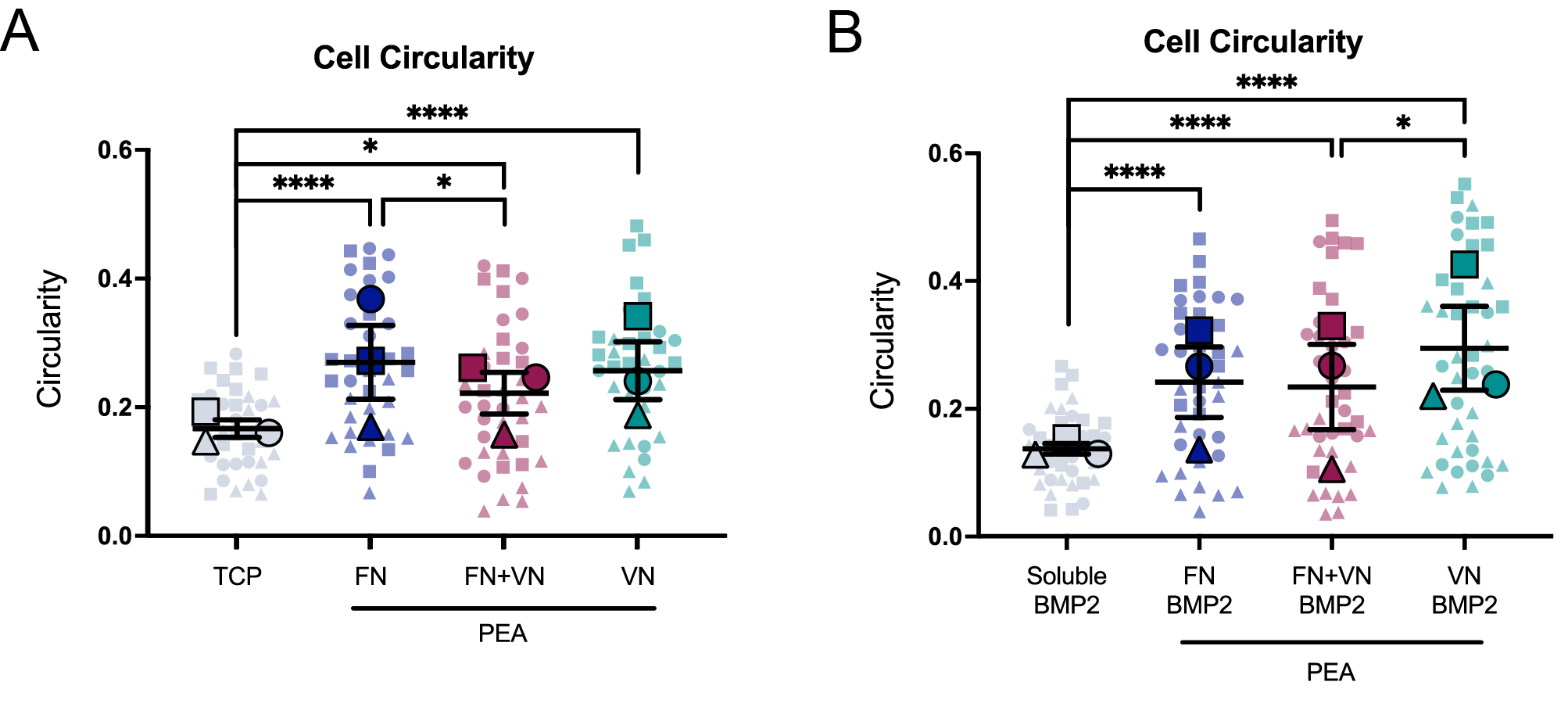


**Figure S5** Cell morphology analysis based on actin and vinculin staining from MSCs on the different substrates. (A), Cell circularity analysis based on cellular actin staining images from MSCs on FN/FN+VN/VN substrates. (B), Cell circularity analysis based on cellular actin staining images from MSCs on FN/FN+VN/VN + BMP2 substrates. Each shape represents a separate cell donor; small shapes represent individual cells and larger shapes show the respective means for each. The scatter plots show mean values and standard mean error. MSCs from 3 donors, and n ≥ 10 cells/donor. Ordinary two-way ANOVA with Tukey’s test for multiple comparisons. *p < 0.05, **p < 0.01, ***p < 0.001, ****p < 0.0001.


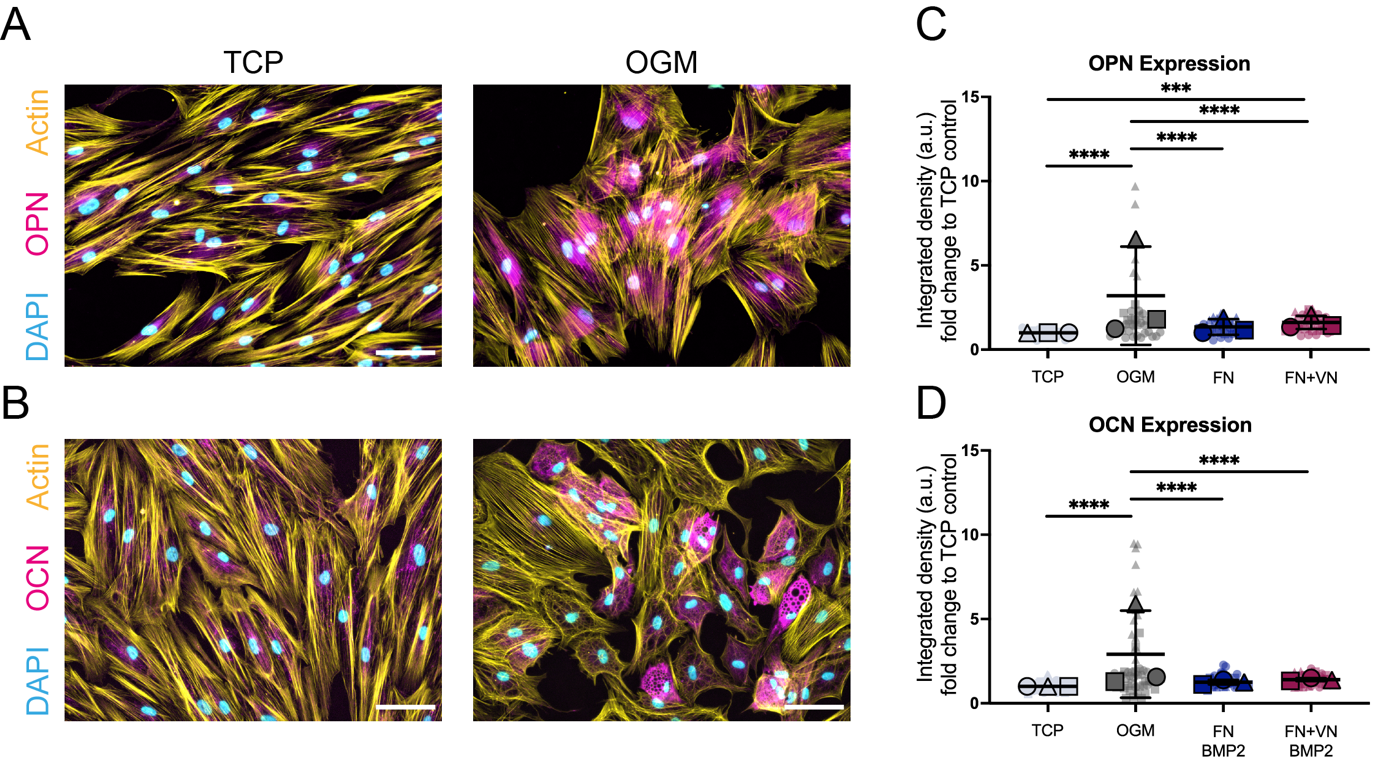


**Figure S6** OPN and OCN staining of MSCs from TCP and OGM groups. (A), Representative images of OPN staining of MSCs from TCP and OGM groups. Scale bar 100 μm. (B), Representative images of OCN staining of MSCs from TCP and OGM groups. Scale bar 100 μm. (C) and (D), Quantification of immunofluorescence images of OPN and OCN staining. Fluorescent intensity fold change to the TCP group was analysed. MSCs from 3 donors. Each shape represents a separate donor; small shapes represent individual cells and larger shapes show the respective means for each. The scatter plots show mean values and standard mean error. Ordinary two-way ANOVA with Tukey’s test was applied for multi-group comparison. *p < 0.05, **p < 0.01, ***p < 0.001, ****p < 0.0001.


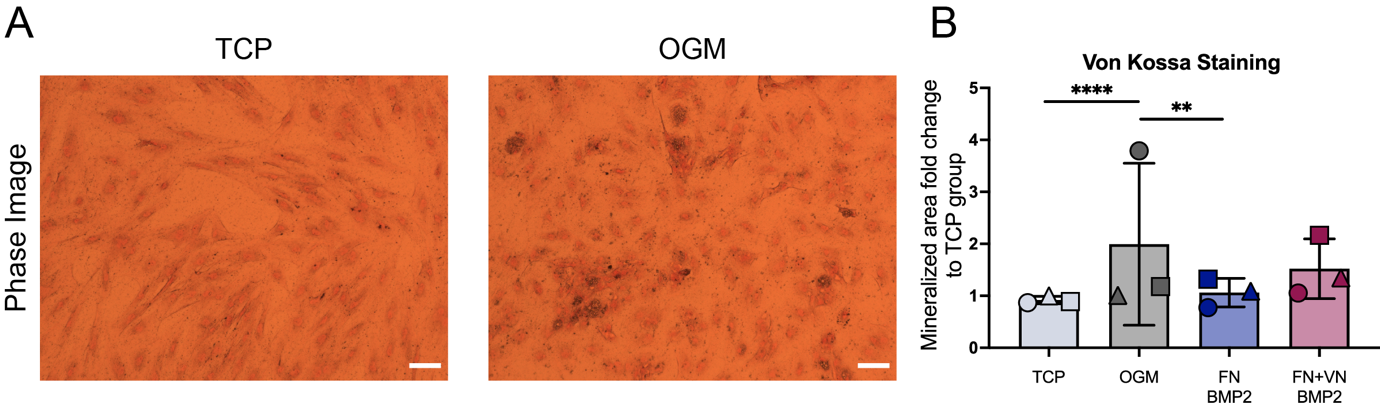


**Figure S7** Von Kossa staining of MSCs from TCP and OGM groups. (A), Representative images of von Kossa staining from MSCs in TCP and OGM groups. Scale bar 100 μm. (B), Quantification of mineralized area based on von Kossa staining of MSCs from TCP and OGM groups. Mineralized area fold change to the FN BMP2 group was analysed. MSCs from 3 donors. Each shape represents the mean value of different donors. The bar plot shows mean values and standard deviation. Ordinary two-way ANOVA with Tukey’s test was applied for multi-group comparison. *p < 0.05, **p < 0.01, ***p < 0.001, ****p < 0.0001.
